# Pharmacokinetics of Vancomycin in Healthy Korean Volunteers and Monte Carlo Simulations to Explore Optimal Dosage Regimens in Patients with Normal Renal Function

**DOI:** 10.3390/antibiotics13100993

**Published:** 2024-10-19

**Authors:** Yong Kyun Kim, Doy Kim, Gaeun Kang, Dae Young Zang, Dong-Hwan Lee

**Affiliations:** 1 Division of Infectious Diseases, Department of Internal Medicine, Hallym University Sacred Heart Hospital, Hallym University College of Medicine, Anyang 14066, Republic of Korea; amoureuxyk@hallym.or.kr; 2 Department of Medicine, Hallym University College of Medicine, Chuncheon 24252, Republic of Korea; lovedoee@naver.com; 3 Division of Clinical Pharmacology, Chonnam National University Hospital, Gwangju 61469, Republic of Korea; bp00092@cnuh.com; 4 Division of Hematology-Oncology, Department of Internal Medicine, Hallym University Sacred Heart Hospital, Hallym University College of Medicine, Anyang 14066, Republic of Korea; fhdzang@hallym.or.kr; 5 Department of Clinical Pharmacology, Hallym University Sacred Heart Hospital, Hallym University College of Medicine, Anyang 14066, Republic of Korea

**Keywords:** vancomycin, population pharmacokinetics, Monte Carlo simulation, normal renal function, healthy, adult

## Abstract

**Background/Objectives**: To date, population pharmacokinetic (PK) studies of vancomycin on healthy Korean adults have not been conducted. This study aimed to investigate the PK properties of vancomycin in healthy volunteers and to identify optimal dosing regimens based on the area under the concentration–time curve (AUC) in adult patients with normal renal function. **Methods**: We conducted a prospective clinical study, analysing PK samples from 12 healthy participants using noncompartmental analysis and non-linear mixed-effects modelling. The population PK parameters derived were employed in Monte Carlo simulations to evaluate the adequacy of the current dosing regimen and to formulate dosing recommendations. **Results**: The PK profiles were optimally described by a two-compartment model, with body weight and age as significant covariates affecting total clearance. The simulations indicated that to achieve a therapeutic target—defined as an AUC at steady-state over 24 h of 400–600 mg·h/L—daily doses ranging from 60 to 70 mg/kg are necessary in adults with normal renal function. **Conclusions**: This study underscores the need to actively adjust dosage and administration based on a vancomycin PK model that adequately reflects the demographic characteristics of patients to meet both safety and efficacy standards.

## 1. Introduction

Hasanpour et al. assessed the global prevalence of methicillin-resistant *Staphylococcus aureus* (MRSA) colonisation among the residents of older adults in care centres in a recent systematic review and meta-analysis. The results indicated that the pooled global prevalence of MRSA was 14.69% in all *S. aureus* blood isolates, with the highest prevalence occurring in the United States at 23.78%, followed by Singapore at 22.72% and Poland at 22.18% [1]. In South Korea, Lee et al. reported an even higher incidence of MRSA in *S. aureus* blood isolates, with 54.3% occurring between May 2016 and April 2017 [2]. This study revealed a marked difference in the prevalence of MRSA, with 69.4% (213/307) of hospital-acquired *S. aureus* infections testing positive for MRSA, compared with 37.5% (104/277) in community-acquired cases.

Vancomycin continues to be the standard treatment for advanced Gram-positive infections worldwide, particularly due to the persistent challenge posed by MRSA and other multidrug-resistant strains in clinical settings. Despite the availability of numerous antibiotics for the treatment of MRSA infections, vancomycin has retained its status as a cornerstone drug in the therapeutic armamentarium. Ju et al. conducted a meta-analysis of 42 randomised controlled trials, which demonstrated the continued importance of vancomycin for treating bloodstream, lung, skin and soft tissue infections [3]. Although newer agents, such as linezolid and daptomycin, show promising efficacy, vancomycin continues to be the standard treatment for certain types of infections due to its proven effectiveness and specific activity against Gram-positive bacteria. This highlights the need to optimise the use of vancomycin to balance efficacy and safety to ensure that it remains a viable treatment option in the face of evolving antibiotic drug resistance.

Recent updates to the consensus guidelines for vancomycin were established based on expert opinion and a large number of research studies. They concluded that trough concentration is not a sufficient surrogate marker for the area under the concentration–time curve (AUC) [4]. The revised guidelines now advocate for monitoring AUC using Bayesian approaches, such as sophisticated population PK models of vancomycin integrated with Bayesian software tools. The guidelines recommend targeting a tailored AUC/MIC ratio of 400–600, based on an assumed vancomycin MIC of 1 mg/L, to optimise clinical outcomes and increase patient safety. Following the publication of the guidelines, numerous studies have evaluated these therapeutic targets [5,6,7,8,9]; however, few studies have assessed the microbiological and clinical endpoints using population PK models under the new guidelines. Therefore, developing models with superior predictive capabilities is necessary to advance this field. During drug development, assessing tolerance and PK in healthy participants is crucial to eliminate the confounding effects of disease and establish the drug’s fundamental characteristics. Similarly, defining standard PK parameters in population-based PK studies not only deepens our understanding of the drug’s behaviour in different populations but also aids in refining models for greater predictive accuracy.

This study aims to develop a population PK model for vancomycin in healthy adults. Using this model, we seek to determine optimal dosing regimens for patients with normal renal function, specifically targeting an AUC/MIC ratio of 400–600 as prescribed by the updated consensus guidelines [4]. This guideline-recommended range is designed to ensure both the safety and efficacy of vancomycin, balancing optimal therapeutic outcomes with minimised risk of nephrotoxicity.

## 2. Results

### 2.1. Participants

The demographic and clinical characteristics of the 12 healthy participants (three females, nine males) are presented in Table 1. The age, height, and weight ranges of the participants were 19–49 years, 159–180 cm, and 53.2–89.4 kg, respectively. The body surface area (BSA) was calculated using the Du Bois Method [10], defined by the following formula: BSA = 0.007185 × height^0.725^ × weight^0.425^. When adjusted for BSA, the estimated glomerular filtration rate (eGFR) based on the modification of diet in renal disease (MDRD) formula was the lowest, whereas the eGFR calculated using the chronic kidney disease epidemiology collaboration (CKD-EPI) formula, which includes creatinine levels and cystatin C, was the highest (Table 1).

### 2.2. Population Pharmacokinetic Analysis

Sixty plasma samples were analysed to characterise the PK profile of vancomycin, which was best described by a two-compartment model (Table 2). The objective function values (OFVs) for the one-, two-, and three-compartment models were −74.864, −162.970, and −163.125, respectively. The selected two-compartment model included parameters such as total clearance (CL), volume of distribution in the central compartment (V1), volume of distribution for the peripheral compartments (V2), and intercompartmental clearance between V1 and V2 (Q2). The parameters of the two-compartment base model are listed in Table S1. The final PK model, with an OFV of –224.007, identified weight and age as significant factors influencing CL, weight as a significant factor influencing Q, and BSA as significantly affecting V2. The detailed covariate screening process is documented in Table S2. The inclusion of the covariates, weight, and age reduced the interindividual variability (IIV) of CL from 28.8% in the basic model to 7.98% in the final model. In the final model, the incorporation of weight decreased the IIV of Q from 33.7% to nearly 0, and the addition of BSA reduced the IIV of V2 from 26.8% to nearly 0. The IIV for V1 was fixed because of its relative standard error (RSE) exceeding 25%.

**Figure 1:**
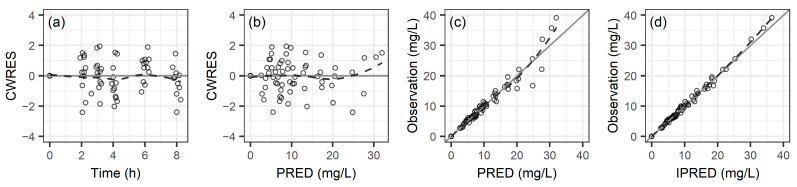
Goodness-of-fit plots for the final pharmacokinetic model of vancomycin: (**a**) conditional weighted residuals (CWRES) vs. time; (**b**) CWRES vs. population predicted concentration (PRED); (**c**) observed concentration vs. PRED; (**d**) observed concentration vs. individual predicted concentration (IPRED). The dashed lines represent smooth curves.

Figure 1 illustrates the goodness-of-fit plots for the final PK model, showing that the distribution of conditional weighted residuals and observed concentrations closely aligns with the *x*-axis and the line of unity (*y* = *x*). This alignment confirms minimal bias in the PK parameters and underscores the model’s suitability. Individual fit plots, displayed in Figure S1, depict the precision in fitting the observed data for vancomycin. The visual predictive check (VPC) for the vancomycin PK model is detailed in Figure S2, where the 10th, 50th, and 90th percentiles of the observed data primarily fall within the 95% confidence interval of the simulated data. This demonstrates the model’s strong predictive capability and its accurate representation of observed concentrations, highlighting its robust predictive accuracy and reliability.

### 2.3. Comparing Noncompartmental Analysis and Population Pharmacokinetics Results

Table 3 presents the results of a descriptive statistical analysis of the PK parameters for each subject, determined using noncompartmental analysis (NCA) and population PK methods. After administering 1 g of vancomycin intravenously, the mean coefficients of variation (CV%) for the area under the plasma concentration–time curve up to the last quantifiable concentration (AUC_last_) and the area under the curve up to infinity (AUC_inf_) were 78.7 (28.2%) mg·h/L and 106 (30.5%) mg·h/L, respectively. When comparing the results analysed using NCA and population PK via a paired t-test, the terminal elimination half-life (t_1/2λz_) and the elimination phase half-life (t_1/2β_) were not statistically different (*p* = 0.9725). However, the total clearance from NCA (CL_NCA_) compared to that from the population PK (CL) and the steady-state volume of distribution from NCA (V_ssNCA_) compared to that from the population PK (V_ss_) showed statistically significant differences (*p* = 0.001747 and 3.173 × 10^−5^, respectively).

### 2.4. Dosage Simulation

**Figure 2:**
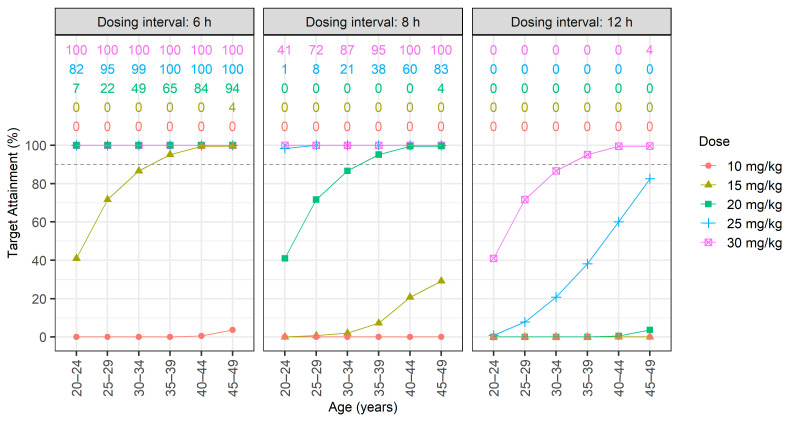
Probability of target attainment (PTA) for the intermittent infusion of vancomycin was assessed across six age groups (from 20–24 to 45–49 years) and administered at five dosage levels (10–30 mg/kg) over three dosing intervals (6, 8, and 12 h). Dots and lines represent therapeutic PTAs achieving an area under curve (AUC) ≥ 400 mg·h/L. The top of the figure represents the proportion of patients with AUC >600 among those achieving AUC ≥ 400. From top to bottom, these correspond to dosages of 30, 25, 20, 15, and 10 mg/kg.

**Figure 3:**
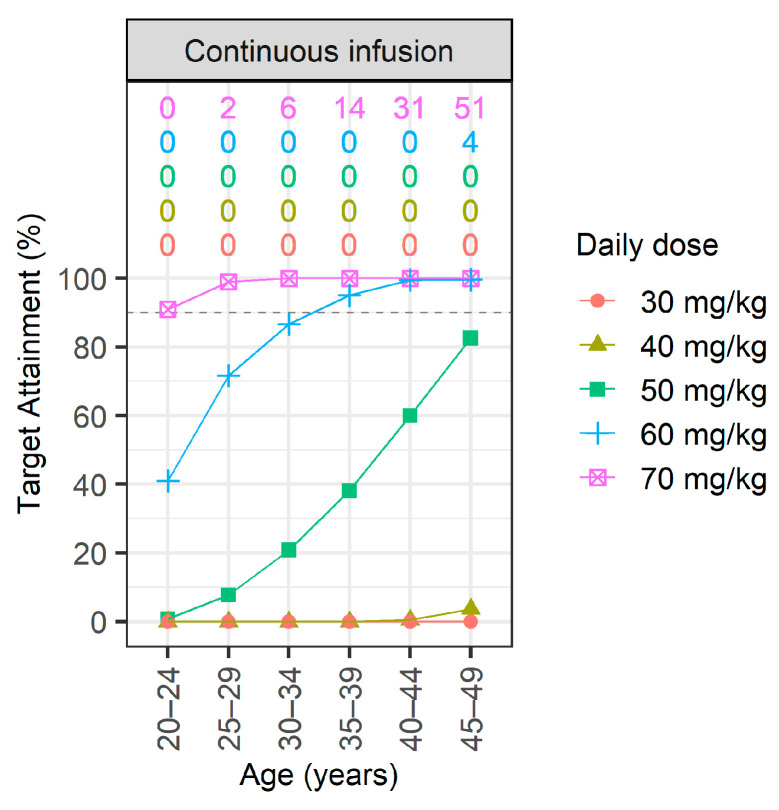
Probability of target attainment (PTA) for continuous vancomycin infusions, as assessed across six age groups (from 20–24 to 45–49 years) at daily doses from 30 to 70 mg/kg. Dots and lines represent therapeutic PTAs achieving an AUC ≥ 400 mg·h/L. The top of the figure represents the proportion of patients with AUC > 600 among those achieving AUC ≥ 400. From top to bottom, these correspond to dosages of 30, 25, 20, 15, and 10 mg/kg.

The therapeutic target was set at an AUC ≥ 400 mg·h/L, and the toxicity target at an AUC > 600 mg·h/L. The PTA for intermittent and continuous infusions is shown in Figure 2 and Figure 3, respectively. Therapeutic PTA percentages are presented as dots and lines, whereas toxicity PTA percentages are displayed numerically at the top of each figure.

**Figure 4:**
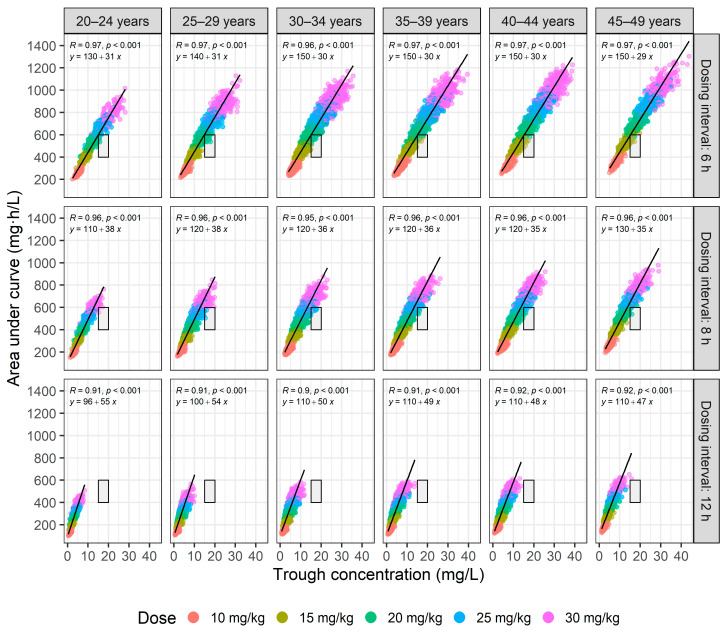
Relationship between the area under the curve (AUC) and trough concentration for intermittent infusions of vancomycin evaluated across six age groups (from 20–24 to 45–49 years) and administered at five dosage levels (10–30 mg/kg) over three dosing intervals (6, 8, and 12 h). Squares indicate 400 mg·h/L ≤ AUC ≤ 600 mg·h/L and 15 mg/L ≤ trough concentration ≤ 20 mg/L. The top of the figure displays the correlation coefficient (R), *p*-value, and the linear regression equation.

For patients with normal renal function, the administration of 20 mg/kg every 8 h in the 35–39 age group resulted in >90% achieving the therapeutic target with a 0% incidence of exceeding an AUC of 600 mg·h/L; however, in the 30–34 age group, the same dosing regimen resulted in <90% PTA. For the 20–24 age group, administering 20 mg/kg every 6 h achieved a therapeutic PTA of 100%, with 6% exceeding an AUC of 600 mg·h/L (Figure 2). The relationship between AUC and trough concentration during intermittent vancomycin administration is shown in Figure 4. Administering doses exceeding 20 mg/kg every 6 or 8 h frequently resulted in trough levels between 15 and 20 mg/L and AUCs > 600 mg·h/L. Conversely, a dose of 30 mg/kg every 12 h typically resulted in trough concentrations below 15 mg/L while still achieving AUCs of at least 400 mg·h/L. This disparity underscores the significant disconnect between AUC and trough concentration.

For patients with normal renal function, continuous infusion is advisable for those aged ≥35 at a daily dose of 60 mg/kg (Figure 3), whereas a dose of 70 mg/kg is recommended for those aged 25–34. The relationship between AUC and steady-state concentration in continuous infusion scenarios, as shown in Figure S3, was confirmed to be directly proportional. This direct proportionality was the result of the steady-state concentration (mg/L) being calculated as AUC divided by 24, with any deviations considered ignorable numerical errors.

## 3. Discussion

In healthy adults, kidney function generally declines with age. The Nijmegen Biomedical Study, involving 3732 adults, demonstrated a gradual decrease in eGFR using the MDRD formula, quantified at a rate of 0.4 mL/min/year [11]. Specifically, eGFR in men typically drops from 100 mL/min/1.73 m^2^ at ages 18–24 to 80 mL/min/1.73 m^2^ by ages 45–49, while in women, it declines from 90 mL/min/1.73 m^2^ to 75 mL/min/1.73 m^2^ over the same age range. A subsequent analysis using the CKD-EPI formula indicated a steeper decline beginning at age 18 [12], suggesting the importance of age-specific thresholds for CKD diagnosis. Delanaye et al. recommended that the eGFR cut-off for diagnosing CKD in individuals over 65 without albuminuria or proteinuria should be adjusted to below 45 mL/min/1.73 m^2^ [13]. A model by Colin et al., which incorporated serum creatinine, body weight, and age, showed a decline in vancomycin clearance starting after age 20, highlighting the importance of understanding drug PK in non-diseased populations for optimal drug management in clinical settings [14]. This understanding is crucial for managing abnormal health conditions by aiming to restore patient conditions to near-normal levels. However, population PK studies of vancomycin in healthy South Korean adults are lacking. This gap prompted our study to develop a robust PK model to improve dosing accuracy, supporting the therapeutic goal of effectively managing drug treatments based on individual PK profiles.

Our study employed a two-compartment model to characterise the pharmacokinetics of vancomycin, revealing that both body weight and age significantly influence drug clearance. We observed a high clearance rate of 8.83 L/h (0.127 L/h/kg) in our cohort of healthy adults with normal renal function, which aligns with findings from 30 population PK models reviewed by Aljutayli et al., showing vancomycin clearance rates ranging from 0.334 to 8.75 L/h (0.0054–0.1279 L/h/kg) [15]. Notable among high-clearance studies, Lin et al. reported a creatinine clearance (CL_CR_) of 104.7 mL/min and vancomycin clearance of 7.56 L/h (0.128 L/h/kg) in 100 post-craniotomy meningitis patients [16]. Li et al. found a vancomycin clearance of 8.75 L/h (0.127 L/h/kg) in 20 postoperative neurosurgical patients, with a serum creatinine level of 0.73 mg/dL [17]. Jarkowski et al. noted a vancomycin clearance of 11.0 L/h (0.127 L/h/kg) in 25 adults with acute myeloid leukaemia, alongside a CL_CR_ of 85.72 mL/min [18]. Additionally, Mali et al. observed in 15 critically ill patients a vancomycin clearance of 8.90 L/h (0.129 L/h/kg) from a single dose and 6.98 L/h (0.101 L/h/kg) at steady-state, with a CLCR of 87.3 mL/min [19]. These results are consistent with our findings, indicating similar vancomycin clearance in healthy adults with normal or near-normal renal function.

Building on this, our final PK model simulated optimal vancomycin dosing for individuals with healthy renal function. The recent guidelines suggest intermittent infusions of 15–20 mg/kg every 8–12 h and continuous infusions starting with a 15–20 mg/kg loading dose, followed by 30–40 mg/kg daily (up to 60 mg/kg), aiming for steady-state concentrations of 20–25 mg/L [4]. We expanded these ranges to assess steady-state AUCs and explored intermittent doses of 10–30 mg/kg at 6, 8, and 12 h intervals, and continuous doses of 30–70 mg/kg daily. We prioritised AUC over the AUC/MIC ratio based on consensus guidelines [4] that typically recommend assuming an empirical vancomycin MIC of 1 mg/L. Calculations confirmed that administering 15 mg/kg every 6 h, 20 mg/kg every 8 h, or 30 mg/kg every 12 h achieves the same 24 h AUC as 60 mg/kg over 24 h. If the target AUC is 400 mg·h/L and vancomycin clearance is 4 L/h, the required daily dose is 1.6 g; this doubles to 3.2 g if the clearance is 8 L/h.

In the present study, the notably high vancomycin clearance necessitates the calculation of significantly larger doses to meet therapeutic targets. Using our final PK model, we determined optimal vancomycin dosing strategies for individuals with normal renal function. Our results are consistent with those of existing studies, which underscores the importance of adjusting dosing regimens when vancomycin clearance is increased. Elder et al. conducted a study of 124 patients with thermal or inhalation injuries and observed varying vancomycin clearance depending on the total body surface area (TBSA) affected by burns [20]. Specifically, vancomycin clearance was 6.9 L/h for TBSA burns < 10%, 8.2 L/h for 10–19%, and 8.9 L/h for 20–29% burns. These findings suggest that a daily dose of 64.7 mg/kg is necessary to achieve a trough concentration of 15–20 mg/L when TBSA exceeds 10%. He et al. treated critically ill patients with a daily dose of 30 mg/kg, observing average AUC values of 232.9 mg·h/L in 139 patients with augmented renal clearance (ARC) and 316.2 mg·h/L in 141 patients without ARC [21]. They reported that ARC patients, with an average CL_CR_ of 180.8 mL/min and a vancomycin clearance of 9.7 L/h, required a daily dose of 46.0 mg/kg to achieve a trough of 10 mg/L. In contrast, non-ARC patients with an average CL_CR_ of 103.4 mL/min and a vancomycin clearance of 6.5 L/h needed a 35.5 mg/kg daily dose to reach the same trough. For severe infections, they recommended 69 mg/kg/day for ARC patients and 53 mg/kg/day for non-ARC patients to achieve a trough of 15 mg/L [21]. Tesfamariam et al. established a population PK model for 219 patients and observed that administering 1 g every 12 h to patients with a CL_CR_ of 150–179 mL/min only achieved a 24 h AUC of 400–650 mg·h/L in 62.56% of the cases [22]. Zhao et al. reported that for 88 patients with ARC with a median renal function of 159 mL/min/1.73 m^2^ and for 326 patients with non-ARC with a median of 78 mL/min/1.73 m^2^, administering 2000 mg/day of vancomycin resulted in median AUC/MIC values of 360.5 and 494.7, respectively [23]. They calculated vancomycin clearances of 5.55 L/h for ARC and 4.04 L/h for non-ARC, which suggests that patients with ARC require an increased dose of 2220 mg/day to achieve an AUC over 400 mg·h/L. Finally, Vu et al. conducted a population PK analysis on 55 ICU patients and recommended dosages of 3.5 g/day for those with a CL_CR_ of 130–180 mL/min and 4.5 g/day for those with a CL_CR_ above 180 mL/min [24]. The corresponding vancomycin clearances were 6.20 L/h for a CL_CR_ of 130 mL/min and 8.61 L/h for a CL_CR_ of 180 mL/min.

Limitations of the present study include a small sample size of 12 individuals, which restricted the identification of significant covariates for PK parameters, including variations in renal function. Additionally, the demographics were limited to a specific age and weight range with generally healthy renal profiles, reducing the variability in renal function metrics (CL_CR_ and eGFR) calculated from different formulas. These factors likely contributed to the non-selection of these metrics as significant covariates in our model. Despite these constraints, our PK model serves as a valuable framework for future studies aimed at refining vancomycin dosing across a more diverse patient population, accommodating a wider range of demographic and physiological variations.

In conclusion, our study established the PK properties of vancomycin in healthy participants using NCA and a population approach. The PK profiles of vancomycin were best described by a two-compartment model, with findings indicating that vancomycin clearance is influenced by body weight and age. While the results from our Monte Carlo simulations suggest that daily doses ranging from 60 to 70 mg/kg may be required to achieve a target AUC of 400–600 mg·h/L in patients with normal renal function, these findings should be approached with caution. Given the limitations of our study, particularly the small sample size and the exclusion of both obese and underweight subjects, these dosage recommendations cannot be generalised to all patient populations without further validation. Therefore, while our study provides valuable insights into the dosing of vancomycin, it underscores the need for additional research to confirm these findings in a broader clinical context.

## 4. Materials and Methods

### 4.1. Participants

This study, approved by the Institutional Review Board of Hallym University Sacred Heart Hospital (IRB No. 2020-12-005), was conducted from August 2021 to July 2022 at the same institution in Anyang, Republic of Korea. As a prospective study, it complied fully with the Declaration of Helsinki and the guidelines of Good Clinical Practice. The inclusion criteria for this study were as follows: (1) individuals aged between 19 and 50 years as of the screening date; (2) individuals with no congenital or chronic diseases and no pathological symptoms or findings upon medical examination; (3) individuals deemed suitable based on health screenings that include medical history, vital signs, physical examination, haematological tests, biochemical blood tests, urinalysis, and other screening tests.

The exclusion criteria were as follows: (1) individuals with clinically significant diseases or history of gastrointestinal, cardiovascular, respiratory, endocrine, hepatobiliary, hematologic-oncologic, musculoskeletal, renal, neurological, psychiatric, immunological, urological, ophthalmological, or otolaryngological disorders, or genetic diseases; (2) individuals with a history of conditions that could affect the absorption, distribution, metabolism, or excretion of drugs, such as gastrointestinal, liver, or kidney diseases; (3) individuals with a known hypersensitivity to vancomycin or a history of such hypersensitivity; (4) individuals with positive serological tests for hepatitis B surface antigen, hepatitis C virus antibodies, human immunodeficiency virus antigen/antibody, or syphilis; (5) pregnant women, nursing mothers, or women with the possibility of becoming pregnant.

### 4.2. Study Design

Participants were administered a 2 h intravenous infusion of 1 g of vancomycin, which was dissolved in 200 mL of normal saline. Venous blood samples (6 mL) were collected into heparinised tubes at pre-determined times: immediately post-dose (2 h) and at 3, 4, 6, and 8 h following the start of infusion. Although no blood samples were collected at time zero, we assumed the concentration to be zero, as the participants were not administered vancomycin before this study. These sampling times were used to evaluate the PK parameters using noncompartmental analysis and population-based PK methods.

### 4.3. Drug Assay

The concentration of vancomycin in plasma was quantified using high-performance liquid chromatography–tandem mass spectrometry (LC-MS/MS). The system comprised a Shimadzu LC-20 setup with a Kinetex XB-C18 analytical column (2.6 μm, 100 × 3.0 mm) and a Gemini C18 guard cartridge (4.0 × 2.0 mm) supplied by Phenomenex, Torrance, California, USA. The mobile phase, consisting of 0.1% formic acid in water and acetonitrile (65:35, *V*/*V*), was delivered at a flow rate of 0.3 mL/min. Data integration was performed using SCIEX Analyst software (version 1.6.3). Detection was achieved on an AB SCIEX 4000 QTRAP system (Sciex, Redwood City, CA, USA) operating in positive ion mode with electrospray ionisation (ESI) and Multiple Reaction Monitoring (MRM), targeting transition masses of m/z 724.9/100.1 for vancomycin and 256.2/167.1 for diphenhydramine (internal standard, IS). Calibration standards were prepared by adding 100 μL of calibration solution and 10 μL of 100 ng/mL diphenhydramine to a microcentrifuge tube, followed by 400 μL acetonitrile for protein precipitation. The sample was vortexed for one minute and centrifuged at 12,000 rpm for two minutes at 4 °C. The supernatant was diluted tenfold with 20 mM ammonium acetate, and 10 μL was injected into the LC-MS/MS system. A similar preparation method was used for the plasma samples, employing diphenhydramine as the internal standard to facilitate protein precipitation. The concentration of vancomycin in plasma was determined by calculating the ratio of the peak area of vancomycin to that of the internal standard and applying a batch-specific calibration curve equation weighted by 1/x.

### 4.4. Population Pharmacokinetic Analysis

PK parameters of vancomycin were evaluated using non-linear mixed-effects models with the NONMEM software package (version 7.5, ICON Clinical Research LLC, North Wales, PA, USA). The estimation of parameters, including fixed and random effects, was conducted using first-order conditional estimation with the interaction method. This enables modelling of the interaction between the IIV in PK parameters and the residual variability (RV) in measured concentrations. For the PK analysis of vancomycin, we used one-, two-, and three-compartment models with the following NONMEM PK model library subroutines: ADVAN1 TRANS2, ADVAN3 TRANS4, and ADVAN11 TRANS4, respectively. Each model adhered to first-order kinetics, except the zero-order infusion processes. Each PK parameter in the model was described by the equation θi = θ × exp (ηi), where θ denotes the typical value of the PK parameter, θi is the individual PK parameter value for each participant, and ηi is a random variable encapsulating IIV. We hypothesised that IIV is normally distributed, with a mean of zero and variance ω^2^. RV was modelled as normally distributed with a mean of zero and a variance σ^2^. Additionally, we evaluated additive, proportional, and combined additive-proportional error models. The methods used to select and validate models encompassed several criteria: variations in NONMEM objective function values (OFVs), the accuracy of parameter estimates as reflected by relative standard errors, goodness-of-fit plots, VPC, and bootstrap evaluations. Notable enhancements in nested model structures were recognised when reductions in OFV exceeded 3.84 (for a one-degree-of-freedom χ^2^ test) or 5.99 (for a two-degrees-of-freedom χ^2^ test), indicating statistical significance with a *p*-value below 0.05. Model validation processes included the creation of conditional weighted residual plots versus time and against population predictions (PRED), in addition to comparisons between observed data and both PRED and individual predictions. We performed VPCs by ensuring observed drug concentrations fell within the 80% prediction intervals derived from 1000 simulations of the model. The variability in the final model predictions was analysed using the median and 95% confidence intervals from 2000 bootstrap replicates. We identified significant covariates influencing pharmacokinetic (PK) parameters through a process of stepwise forward selection and backward elimination, with inclusion thresholds of *p* < 0.01 (ΔOFV < −6.635) and exclusion thresholds of *p* < 0.001 (ΔOFV > 10.83) for single degree-of-freedom tests. Covariates were assessed for both their statistical and clinical relevance, taking into account demographic factors (such as gender, age, weight, height, body mass index, and body surface area) and biochemical markers (including serum protein, albumin, creatinine, and cystatin C levels). The influence of renal clearance on vancomycin elimination was analysed using the Cockcroft-Gault [25], MDRD [26], and CKD-EPI equations [27]. We utilised Perl-speaks-NONMEM (PSN, version 5.3.1, [https://uupharmacometrics.github.io/PsN], accessed on 13 April 2023) for covariate identification, VPC execution, and nonparametric bootstrapping to evaluate model robustness. Data analysis and graphical outputs were conducted using the R programming environment (version 4.4.0, [www.rproject.org], Vienna, Austria, accessed on 6 June 2024).

### 4.5. Noncompartmental Analysis

An NCA was conducted using the R programming language and the NonCompart package [28] to analyse the plasma concentration–time profiles of vancomycin. The PK parameters evaluated included the maximum observed plasma concentration (C_max_), time to last measurable concentration (T_last_), and the concentration at T_last_ (C_last_). We calculated AUC_last_ and AUC_inf_, and the area under the first moment curve (AUMC) from zero to T_last_ (AUMC_last_) and extrapolated to infinity (AUMC_inf_). Other parameters determined were mean residence time to infinity (MRT_inf_), CL_NCA_, V_ssNCA_, and the t_1/2λz_. The AUC_last_ and AUMC_last_ were derived using the linear-up and log-down trapezoidal methods. AUC_inf_ was estimated by extrapolating AUC_last_ with the addition of C_last_/λ_z_, where λ_z_ is the terminal elimination rate constant derived from log-linear regression of terminal phase plasma concentrations. The calculation of AUMC_inf_ included the formula: AUMC_last_ + (T_last_ × C_last_)/λ_z_ + C_last_/λ_z_^2^. Furthermore, MRT_inf_ was calculated as AUMC_inf/_AUC_inf_ minus half the infusion time, CL_NCA_ was derived from the dose divided by AUC_inf_, V_zNCA_ was calculated as CL_NCA_ divided by λ_z_, V_ssNCA_ as the product of MRT_inf_ and CL_NCA_, and t_1/2λz_ as the natural logarithm of 2 divided by λ_z_.

### 4.6. Dosage Simulation

To develop vancomycin dosage recommendations for patients with normal renal function, we used Monte Carlo simulations based on our final PK model. The primary PK/PD index for vancomycin is the AUC/MIC ratio, which guides dosing strategies. The AUC is defined as the area under the vancomycin concentration–time curve at steady-state over 24 h. Based on current consensus guidelines, an AUC of 400–600 mg·h/L is recommended for the effective treatment of serious MRSA infections when the MIC is ≤1 mg/L. We performed a series of simulations to assess the probability of achieving this therapeutic target (AUC ≥ 400 mg·h/L) and to evaluate potential nephrotoxicity (AUC > 600 mg·h/L). The simulations included 2000 virtual participants, and CL was modelled using the parameter estimates of our final PK model. Individual variability and continuous covariates are represented as log-normally distributed random variables across a demographic range. The AUC was calculated as dose/CL. Two analyses were performed to assess the potential therapeutic efficacy of vancomycin. The first analysis calculated the AUC for 2000 participants at five doses (ranging from 10 to 30 mg/kg in 5 mg/kg increments) and three dosing intervals (6, 8, and 12 h), with the probability of target attainment (PTA) compared among six age groups (20–24, 25–29, 30–34, 35–39, 40–44, and 45–49 years). The first analysis also evaluated the relationship between AUC and trough concentration. The second analysis involved AUC calculations for 1000 participants receiving five doses (30, 40, 50, 60, and 70 mg/kg) through continuous intravenous infusion, with PTA comparisons for the six age groups. In addition, the analysis further examined the relationship between AUC and steady-state concentration.

## Figures and Tables

**Table 1:** Subject characteristics.

Parameters	Mean (CV%)	Median (1Q–3Q)
Demographic characteristics		
Age, years	35.9 (29.4)	38.5 (31.5–43.3)
Height, cm	170 (4.16)	172 (164–175)
Weight, kg	70.0 (18.0)	69.5 (60.7–78.2)
Body surface area, m^2^	1.81 (10.1)	1.80 (1.70–1.92)
Body mass index, kg/m^2^	24.0 (13.4)	23.3 (21.4–26.5)
Laboratory characteristics		
Protein, g/dL	7.53 (6.00)	7.45 (7.30–7.55)
Albumin, g/dL	4.80 (3.97)	4.80 (4.68–5.00)
Cystatin C, mg/dL	0.717 (21.1)	0.720 (0.595–0.805)
Creatinine, mg/dL	0.866 (22.2)	0.895 (0.753–1.01)
Blood urea nitrogen, mg/dL	13.4 (23.9)	12.8 (11.1–14.5)
Alanine aminotransferase, U/L	14.5 (39.7)	12.0 (10.0–20.0)
Aspartate aminotransferase, U/L	18.0 (21.6)	18.5 (17.0–20.5)
Gamma-glutamyl transferase, U/L	20.8 (61.8)	15.5 (14.0–22.0)
Renal functions		
CL_CR_, CG (mL/min) ^a^	117 (29.1)	113 (102–124)
CL_CR_, normalised CG (mL/min/1.73 m^2^) ^b^	112 (27.1)	106 (102–116)
eGFR, MDRD (mL/min/1.73 m^2^) ^c^	98.3 (21.9)	96.9 (84.9–104)
eGFR, CKD-EPI_CR_ (mL/min/1.73 m^2^) ^d^	109 (13.5)	110 (98.7–116)
eGFR, CKD-EPI_CR-CYS_ (mL/min/1.73 m^2^) ^e^	118 (13.0)	120 (111–125)
eGFR, adjusted MDRD (mL/min) ^f^	102 (22.6)	99.1 (93.3–110)
eGFR, adjusted CKD-EPI_CR_ (mL/min) ^f^	113 (14.9)	113 (104–121)
eGFR, adjusted CKD-EPI_CR-CYS_ (mL/min) ^f^	123 (14.9)	129 (115–134)

CV, coefficient of variation; IQR, interquartile range; CL_CR_, creatinine clearance; eGFR, estimated glomerular filtration rate; MDRD, modification of diet in renal disease; CKD-EPI, chronic kidney disease epidemiology collaboration; CR, creatinine; CYS, cystatin C; min, the minimum of (CR or CYS)/number and 1; max, the maximum of (CR or CYS)/number and 1. ^a^ CL_CR_ = (140 − Age) × weight/CR × 72 (×0.85 if female). ^b^ The normalised CL_CR_ by Cockcroft–Gault equations are CL_CR_ = CL_CR_, CG/BSA × 1.73 m^2^. ^c^ eGFR = 175 × CR^−1.154^ × Age^−0.203^ (×0.742 if female). ^d^ eGFR (female) = 142 × min (CR/0.7,1)^−0.241^ × max (CR/0.7,1)^−1.200^ × 0.9938^Age^ × 1.012. eGFR (male) = 142 × min (CR/0.9,1)^−0.302^ × max (CR/0.9,1)^−1.200^ × 0.9938^Age^. ^e^ eGFR (female) = 135 × min (CR/0.7,1)^−0.219^ × max (CR/0.7,1)^−0.544^ × min (CYS/0.8,1)^0.323^ × max (CYS/0.8,1)^−0.778^ × 0.9961^Age^ × 0.963. eGFR (male) = 135 × min (CR/0.9,1)^−0.144^ × max (CR/0.9,1)^−0.544^ × min (CYS/0.8,1)^0.323^ × max (CYS/0.8,1)^−0.778^ × 0.9961^Age^. ^f^ The adjusted eGFR by MDRD and CKD-EPI equations are eGFR = eGFR (MDRD or CKD-EPI)/1.73 m^2^ × BSA.

**Table 2:** Parameter estimates and bootstrap medians (95% confidence intervals) for the final pharmacokinetic model of vancomycin in 12 healthy adult participants.

Parameter	Estimates	RSE (%) [Shrinkage, %]	Bootstrap Median (95% CI)
Structural model			
CL = θ_1_ × (WT/69.45) ^θ2^ × (AGE/38.5) ^θ3^			
θ_1_ (L/h)	8.83	3.16	8.78 (8.14–9.6)
θ_2_	1.28	8.13	1.30 (0.955–1.58)
θ_3_	−0.377	19.2	−0.376 (−0.584–−0.129)
V1 = θ_4_			
θ_4_ (L)	12.9	26.4	12.8 (3.94–19.6)
Q = θ_5_ × (WT/69.45) ^θ6^			
θ_5_ (L/h)	20.2	10.0	20.4 (16.8–28.2)
θ_6_	2.38	17.6	2.31 (1.36–3.35)
V2 = θ_7_ × (BSA/1.80) ^θ8^			
θ_7_ (L)	27.4	4.17	27.8 (25.0–31.0)
θ_8_	3.09	11.7	3.13 (1.79–3.73)
Interindividual variability			
CL (%)	7.98	18.3 [9.92]	6.90 (1.65–9.52)
V1 (%)	47.4 ^f^	[16.6]	
Residual variability			
Proportional error (%)	6.94	9.21 [13.3]	6.79 (5.22–8.02)

RSE, relative standard error; CI, confidence interval; CL, total clearance; V1, central volume of distribution; V2, volume of distribution for the peripheral compartment; Q, intercompartmental clearance between V1 and V2; WT, weight; BSA, body surface area; ^f^, fixed.

**Table 3:** Descriptive statistics of individual pharmacokinetic parameters from noncompartmental and population PK analysis.

Parameters	Unit	Mean (CV%)	Median (IQR)
NCA results			
C_max_	mg/L	23.4 (34.4)	20.8 (17.0–27.1)
C_last_	h	4.68 (33.9)	4.65 (3.13–5.90)
T_last_	mg/L	7.99 (1.93)	7.99 (7.88–8.10)
AUC_last_	mg·h/L	78.7 (28.2)	73.6 (58.5–90.4)
AUC_inf_	mg·h/L	106 (30.5)	97.1 (76.1–140)
AUMC_last_	mg·h^2^/L	289 (26.7)	279 (217–341)
AUMC_inf_	mg·h^2^/L	667 (40.0)	587 (464–807)
MRT_inf_	h	5.12 (18.9)	4.98 (4.46–5.60)
CL_NCA_	L/h/kg	0.146 (19.8)	0.135 (0.128–0.160)
V_zZNCA_	L/kg	0.793 (15.4)	0.806 (0.701–0.873)
V_ssNCA_	L/kg	0.730 (15.7)	0.714 (0.650–0.802)
t_1/2λz_	h	3.87 (21.3)	3.58 (3.34–4.25)
Population PK results			
CL	L/h/kg	0.135 (17.0)	0.131 (0.117–0.144)
V_C_	L/kg	0.211 (48.0)	0.163 (0.144–0.251)
V_ss_	L/kg	0.611 (16.5)	0.593 (0.565–0.650)
AUC_tau_	mg·h/L	113 (28.9)	111 (81.9–137)
t_1/2α_	h	0.270 (42.7)	0.259 (0.177–0.300)
t_1/2β_	h	3.88 (12.5)	3.84 (3.63–4.26)

CV, coefficient of variation; IQR, interquartile range; C_max_, maximum observed plasma concentration; T_last_, time of last measurable concentration; C_last_, concentration corresponding to T_last_; AUC_last_, area under the plasma concentration–time curve (AUC) from the start of dosing to the last quantifiable concentration; AUC_inf_, AUC from the start of dosing to infinity; AUMC_last_, area under the first moment curve (AUMC) from 0 h to the T_last_; AUMC_inf_, AUMC extrapolated to infinity, based on the last observed concentration; MRT_inf_, mean residence time from 0 h to infinite; CL_NCA_, total body clearance determined by NCA; V_zNCA_, volume of distribution (Vd) determined by NCA; V_ssNCA_, steady-state Vd determined by NCA; t_1/2λz_, terminal elimination half-life. Formulas: AUC_inf_, AUC_last_ + C_last_/λ_z_; AUMC_inf_, AUMClast + (T_last_ × Clast)/λ_z_ + Clast/λ_z_^2^; MRT_inf_, AUMC_inf_/AUC_inf_ − infusion time/2; CL_NCA_, dose/AUC_inf_; V_zNCA_, CL_NCA_/λ_z_; V_ssNCA_, MRT_inf_ × CL_NCA_; t_1/2λz_, ln (2)/λ_z_; CL, total clearance; V_C_, central volume of distribution; V_ss_, steady-state volume of distribution; AUC_tau_, dose/CL; t_1/2α_, distribution phase half-life; t_1/2β_, elimination phase half-life.

## Data Availability

The datasets generated and/or analysed during the current study are available from the corresponding author upon reasonable request.

## Supplementary Materials

The following supporting information can be downloaded at: https://www.mdpi.com/article/10.3390/antibiotics13100993/s1, Table S1. Parameter estimates for the base pharmacokinetic model of meropenem in 12 healthy adult participants. Table S2. Stepwise covariate selection process: forward selection (p-value = 0.01, OFV difference > 6.635, degree of freedom = 1) and backward elimination (p-value = 0.001, OFV difference < −10.83, degree of freedom = 1.) Figure S1. Individual fit plots of vancomycin (a) normal scale, (b) semi-log scale: closed circles, observed concentrations; solid line, individual-predicted concentrations; dotted line, population-predicted concentrations. Figure S2. Visual predictive check from simulated concentrations of 1000 virtual datasets of vancomycin: closed circles, observed concentrations; solid lines, 10th, 50th, and 90th percentiles of observations; dashed lines, 10th, 50th, and 90th percentiles of simulated concentrations; and shaded areas, 95% confidence intervals for the 10th, 50th, and 90th percentiles of simulated concentrations. Figure S3. Relationship between area under the curve (AUC) and trough concentration for continuous infusions of vancomycin, evaluated across six age groups (20–24 to 45–49 years) at daily doses from 30 to 70 mg/kg. Squares indicate where 400 ≤ AUC ≤ 600 mg·h/L and 20 ≤ trough concentration ≤ 25 mg/L. The top of the figure displays the correlation coefficient (R), *p*-value, and the linear regression equation.
