# Supplementary material for: Pharmacokinetics of Vancomycin in Healthy Korean Volunteers and Monte Carlo Simulations to Explore Optimal Dosage Regimens in Patients with Normal Renal Function
Source: Antibiotics (Basel). 2024 Oct 19;13(10):993. doi: 10.3390/antibiotics13100993 (PMC11504268; doi:10.3390/antibiotics13100993)
Supplement: Supplementary file 1 [file antibiotics-13-00993-s001.zip › antibiotics-3185709-supplementary.pdf]

Table S1. Parameter estimates for the base pharmacokinetic model of meropenem in 12 healthy adult participants

Parameter	Estimates	RSE (%) [Shrinkage, %]
Structural model		
CL (L/h)	9.11	8.74
V1 (L)	15.1	19.7
Q (L/h)	17.8	13.8
V2 (L)	26.2	9.63
Interindividual variability		
CL (%)	28.8	9.34 [1.89]
V1 (%)	55.2	24.8 [18.1]
Q (%)	33.7	18.9 [29.1]
V2 (%)	26.8	20.6 [14.1]
Residual variability		
Proportional error (%)	6.90	12.3 [27.5]

RSE, relative standard error; CI, confidence interval; CL, total clearance; V1, central volume of distribution; V2, volume of distribution for the peripheral compartment; Q, inter-compartmental clearance between V1 and V2.

Table S2. Stepwise covariate selection process: forward selection (p-value = 0.01, OFV difference > 6.635, degree of freedom = 1) and backward elimination (p-value = 0.001, OFV difference < -10.83, degree of freedom = 1)

Parameter	Covariate	Base OFV	New OFV	OFV difference	P-value
Forward step 1					
CL	BMI	-162.9470	-172.4260	9.4791	0.0021
CL	BSA	-162.9470	-176.8238	13.8768	0.0002
CL	$eGFR_{CE\_CRCYS2}$ [a]	-162.9470	-171.6349	8.6880	0.0032
CL	$eGFR_{CE\_CR2}$ [b]	-162.9470	-171.5227	8.5758	0.0034
CL	$eGFR_{CE\_CYS2}$ [c]	-162.9470	-171.2118	8.2648	0.0040
CL	CL_{CR_CG} [d]	-162.9470	-172.4631	9.5162	0.0020
CL	Height	-162.9470	-169.6265	6.6795	0.0098
CL	Weight	-162.9470	-177.3217	14.3747	0.0002
Q	BMI	-162.9470	-175.2952	12.3483	0.0004
Q	BSA	-162.9470	-174.2060	11.2590	0.0008
Q	Weight	-162.9470	-175.8597	12.9128	0.0003
V2	BMI	-162.9470	-170.3979	7.4509	0.0063
V2	BSA	-162.9470	-182.8210	**19.8741**	0.0000
V2	Height	-162.9470	-176.3014	13.3544	0.0003
V2	Weight	-162.9470	-181.0272	18.0802	0.0000
Forward step 2					
CL	BMI	-182.8210	-191.4654	8.6444	0.0033
CL	BMI	-182.8210	-195.1683	12.3473	0.0004
CL	$eGFR_{CE\_CRCYS2}$ [a]	-182.8210	-191.3072	8.4861	0.0036
CL	$eGFR_{CE\_CR2}$ [b]	-182.8210	-192.2315	9.4105	0.0022
CL	$eGFR_{CE\_CYS2}$ [c]	-182.8210	-190.4497	7.6287	0.0057
CL	CL_{CR_CG}	-182.8210	-192.7896	9.9686	0.0016
CL	$eGFR_{MDRD2}$ [e]	-182.8210	-189.7587	6.9377	0.0084
CL	Weight	-182.8210	-195.6102	12.7891	0.0003
Q	BMI	-182.8210	-194.0803	11.2593	0.0008
Q	BSA	-182.8210	-194.8653	12.0442	0.0005
Q	Weight	-182.8210	-196.3560	**13.5350**	0.0002
Forward step 3					
CL	BMI	-196.3560	-205.9978	9.6418	0.0019
CL	BSA	-196.3560	-210.7633	14.4073	0.0001
CL	$eGFR_{CE\_CRCYS2}$ [a]	-196.3560	-204.6756	8.3196	0.0039
CL	$eGFR_{CE\_CR2}$ [b]	-196.3560	-205.4963	9.1403	0.0025
CL	$eGFR_{CE\_CYS2}$ [c]	-196.3560	-204.0901	7.7341	0.0054
CL	CL_{CR_CG}	-196.3560	-206.0667	9.7107	0.0018
CL	Height	-196.3560	-203.3174	6.9614	0.0083
CL	Weight	-196.3560	-211.2074	**14.8514**	0.0001
Forward step 4					
CL	Age	-211.2074	-224.0071	**12.7997**	0.0003
Backward step 1					
CL	Age	-224.0071	-211.2074	-12.7997	0.0003
CL	Weight	-224.0071	-200.4014	-23.6056	0.0000

Q	Weight	-224.0071	-206.7403	-17.2667	0.0000
V2	BSA	-224.0071	-203.5727	-20.4344	0.0000

OFV, objective function value; BMI, body mass index; BSA, body surface area; CL_{CR_CG}, creatinine clearance by Cockcroft-Gault equation; eGFR, estimated glomerular filtration rate; MDRD, modification of diet in renal disease; CKD-EPI, chronic kidney disease epidemiology collaboration; CR, creatinine; CYS, cystatin C; min, the minimum of (CR or CYS)/number and 1; max, the maximum of (CR or CYS)/number and 1. [a] eGFR (mL/min) [female] = $135 \times \min(CR/0.7,1)^{-0.219} \times \max(CR/0.7,1)^{-0.544} \times \min(CYS/0.8,1)^{0.323} \times \max(CYS/0.8,1)^{-0.778} \times 0.9961^{Age} \times 0.963 / 1.73\ m^2 \times BSA$. eGFR (mL/min) [male] = $135 \times \min(CR/0.9,1)^{-0.144} \times \max(CR/0.9,1)^{-0.544} \times \min(CYS/0.8,1)^{0.323} \times \max(CYS/0.8,1)^{-0.778} \times 0.9961^{Age} / 1.73\ m^2 \times BSA$. [b] eGFR (mL/min) [female] = $142 \times \min(CR/0.7,1)^{-0.241} \times \max(CR/0.7,1)^{-1.200} \times 0.9938^{Age} \times 1.012 / 1.73\ m^2 \times BSA$. eGFR (mL/min) [male]= $142 \times \min(CR/0.9,1)^{-0.302} \times \max(CR/0.9,1)^{-1.200} \times 0.9938^{Age} / 1.73\ m^2 \times BSA$. [c] eGFR (mL/min) = $133 \times \min(CYS/0.7,1)^{-0.499} \times \max(CYS/0.7,1)^{-1.328} \times 0.9962^{Age}$ (× 0.932 if female) / $1.73\ m^2 \times BSA$. [d] CL_{CR} (mL/min)= (140−Age) × weight/CR × 72 (× 0.85 if female). [e] eGFR (mL/min) = $175 \times CR^{-1.154} \times Age^{-0.203}$ (× 0.742 if female) / $1.73\ m^2 \times BSA$.

Supplementary figures

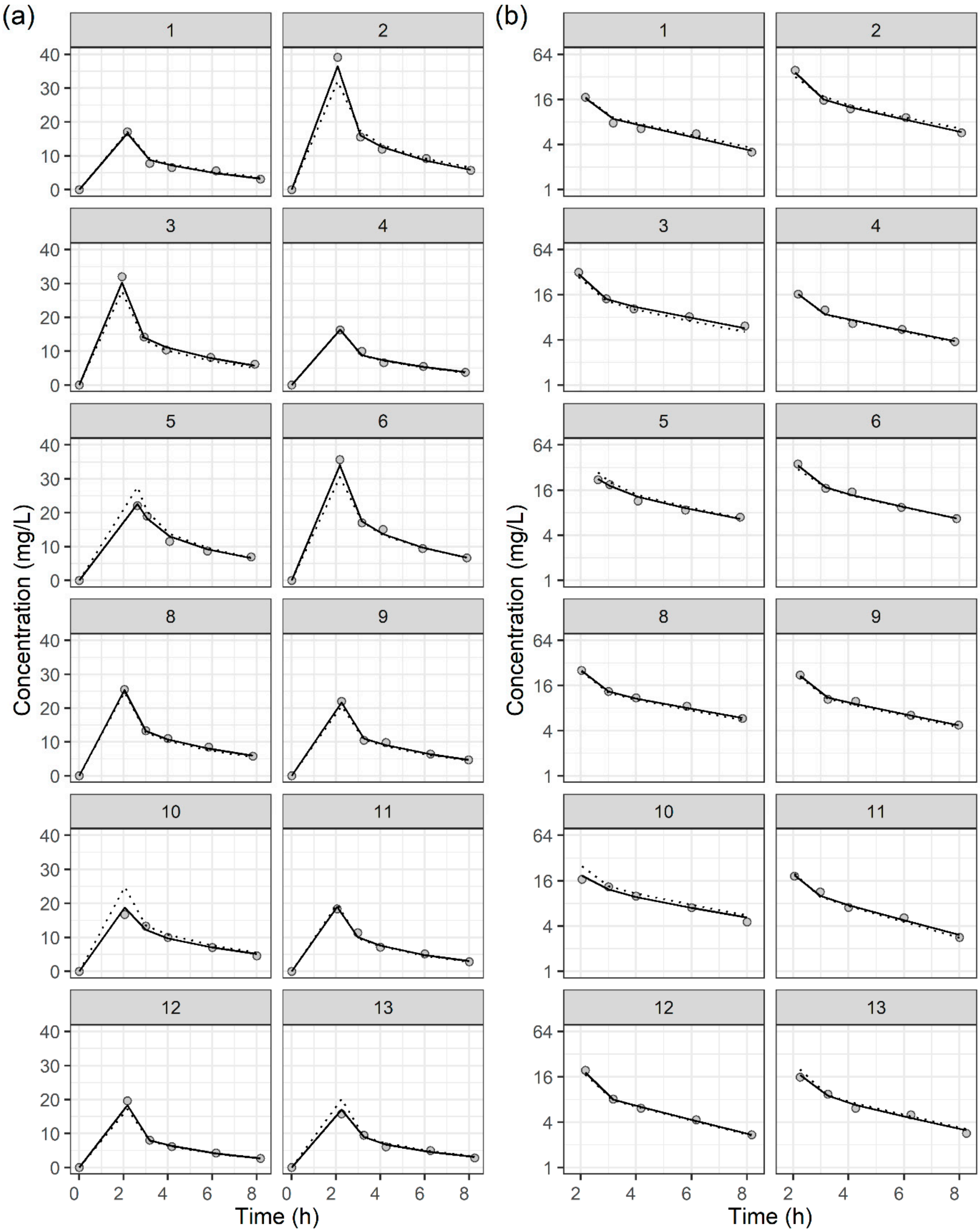

Figure S1. Individual fit plots of vancomycin (a) Normal scale, (b) semi-log scale: closed circles, observed concentrations; solid line, individual-predicted concentrations; dotted line, population-predicted concentrations.

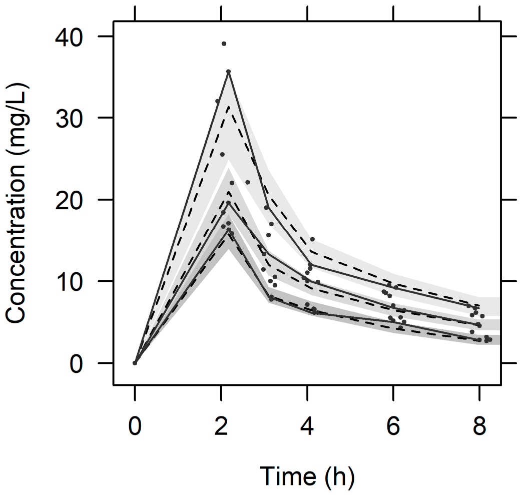

Figure S2. Visual predictive check from simulated concentrations of 1,000 virtual datasets of vancomycin: closed circles, observed concentrations; solid lines, 10th, 50th and 90th percentiles of observations; dashed lines, 10th, 50th and 90th percentiles of simulated concentrations; and shaded areas, 95% confidence intervals for the 10th, 50th, and 90th percentiles of simulated concentrations.

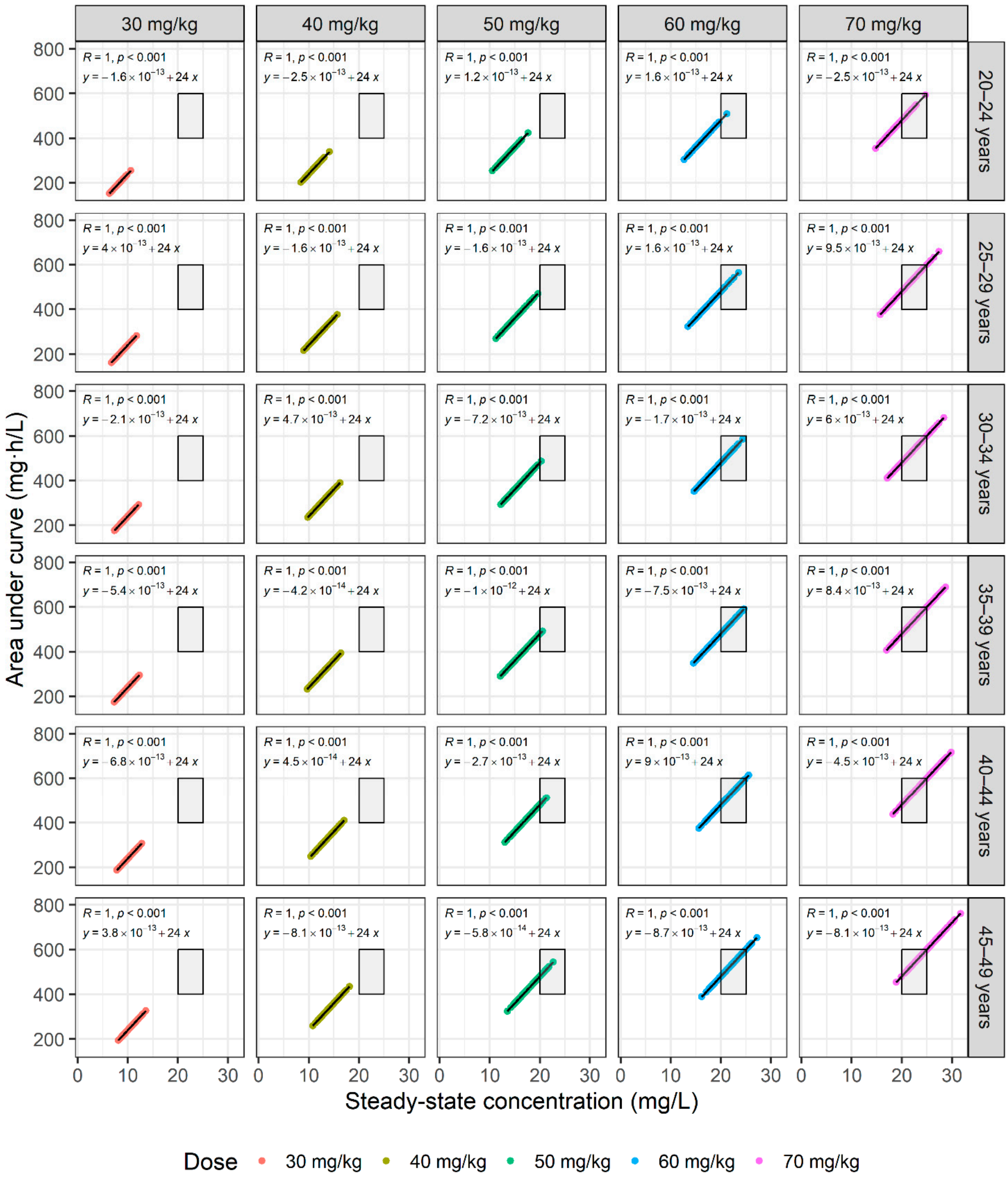

Figure S3. Relationship between area under the curve (AUC) and trough concentration for continuous infusions of vancomycin, evaluated across six age groups (20–24 to 45-49 years) at daily doses from 30 to 70 mg/kg. Squares indicate where 400 ≤ AUC ≤ 600 mg·h/L and 20 ≤ trough concentration ≤ 25 mg/L. The top of the figure displays the correlation coefficient (R), p-value, and the linear regression equation.
